# Amorphous Solid Dispersions Layered onto Pellets—An Alternative to Spray Drying?

**DOI:** 10.3390/pharmaceutics15030764

**Published:** 2023-02-24

**Authors:** Marius Neuwirth, Sebastian K. Kappes, Michael U. Hartig, Karl G. Wagner

**Affiliations:** Department of Pharmaceutics, University of Bonn, 53121 Bonn, Germany

**Keywords:** amorphous solid dispersions, fluid bed, spray drying, pellet coating, supersaturation, release kinetics

## Abstract

Spray drying is one of the most frequently used solvent-based processes for manufacturing amorphous solid dispersions (ASDs). However, the resulting fine powders usually require further downstream processing when intended for solid oral dosage forms. In this study, we compare properties and performance of spray-dried ASDs with ASDs coated onto neutral starter pellets in mini-scale. We successfully prepared binary ASDs with a drug load of 20% Ketoconazole (KCZ) or Loratadine (LRD) as weakly basic model drugs and hydroxypropyl-methyl-cellulose acetate succinate or methacrylic acid ethacrylate copolymer as pH-dependent soluble polymers. All KCZ/ and LRD/polymer mixtures formed single-phased ASDs, as indicated by differential scanning calorimetry, X-ray powder diffraction and infrared spectroscopy. All ASDs showed physical stability for 6 months at 25 °C/65% rH and 40 °C/0% rH. Normalized to their initial surface area available to the dissolution medium, all ASDs showed a linear relationship of surface area and solubility enhancement, both in terms of supersaturation of solubility and initial dissolution rate, regardless of the manufacturing process. With similar performance and stability, processing of ASD pellets showed the advantages of a superior yield (>98%), ready to use for subsequent processing into multiple unit pellet systems. Therefore, ASD-layered pellets are an attractive alternative in ASD-formulation, especially in early formulation development at limited availability of drug substance.

## 1. Introduction

Amorphous solid dispersions (ASDs) are an established and well-described technique for improving the oral bioavailability of poorly soluble active pharmaceutical ingredients (APIs). It is mainly used for APIs classified as class II drugs in the biopharmaceutical classification system (BCS), which means that they are poorly soluble in water but quickly penetrate biological barriers [1,2,3,4]. The production of ASDs can generally be classified into two types of processes: solvent-based methods or fusion methods. Solvent-based processes including spray drying (SD), fluid bed granulation/layering, co-precipitation, electrospinning, supercritical fluid impregnation and vacuum drum drying [5,6,7,8,9].

A majority of the formulations currently on the market are based on solvent-based processes, and are manufactured using SD [10]. To our knowledge, the only formulation approved in the EU where an itraconazole-ASD has been coated onto pellets is Sporanox^®^ from Jannssen Pharmaceutica (Beerse, Belgium) [11,12] and its generic successors. Although SD can theoretically be understood as a continuous process, the yield and flow properties of the fine powder obtained are not sufficient to implement it economically, together with other manufacturing/downstream processes within a continuous manufacturing line [13]. In order to transfer these fine particles into free-flowing agglomerates, further downstream processes such as, e.g., roller compaction have to be performed subsequently [14]. This further processing poses an increased risk of recrystallization of the amorphous system, due to compaction-induced phase changes [15]. Consequently, amorphous intermediates should be subjected to as few processing steps as possible. Especially in early development, the limited yield in small scale spray drying, combined with the above-mentioned downstream processing, demands high efforts in development resources.

In contrast to the genuine fine particles of SD processes, pellets exhibit beneficial properties, such as free flow and narrow particle size distribution. With that, pellets coated with an ASD are suitable for direct capsule filling as well as for compression into multi-particulate systems such as multiple-unit pellet systems (MUPS) [16,17,18] without the demanding downstream processing related to SD powders. Therefore, pellet formulations are potentially more physically stable compared to spray-dried particles given the reduced development efforts. Moreover, ASD pellets can be used in personalized medicine, as capsules or drinking straws could be filled with individual doses, which is relatively easy, compared to, e.g., 3D-printing using ASD-filaments.

Despite the above-mentioned potential advantages of ASD-layered pellets over SD, the potential does not reflect the market situation of ASDs. In the past few years, a number of papers have been published addressing the layering of ASDs onto various starter cores (mesoporous silica [19] or sugar beads [20,21,22,23]) using pH-independent soluble polymers (povidone, copovidone, hydroxypropyl-methyl-cellulose) as matrix. However, neither of the above-mentioned publications directly compared the ASD performance between the two processes of SD and ASD pellet coating (PC), nor used pH-dependent soluble (gastro resistant) polymers as ASD matrix. Therefore, we intended to systematically investigate the feasibility of producing ASDs coated onto pellets with a lab scale fluid bed system and to compare these pellets with ASD granules prepared by dry granulated SD powders. For this purpose, ASDs were prepared by SD, and cellulose starter-pellets were coated with an ASD of the same composition. Subsequently, the ASDs were analyzed, with focused attention to their particle size distribution, shape characteristics, physical stability, and dissolution behavior.

**Figure 1:**
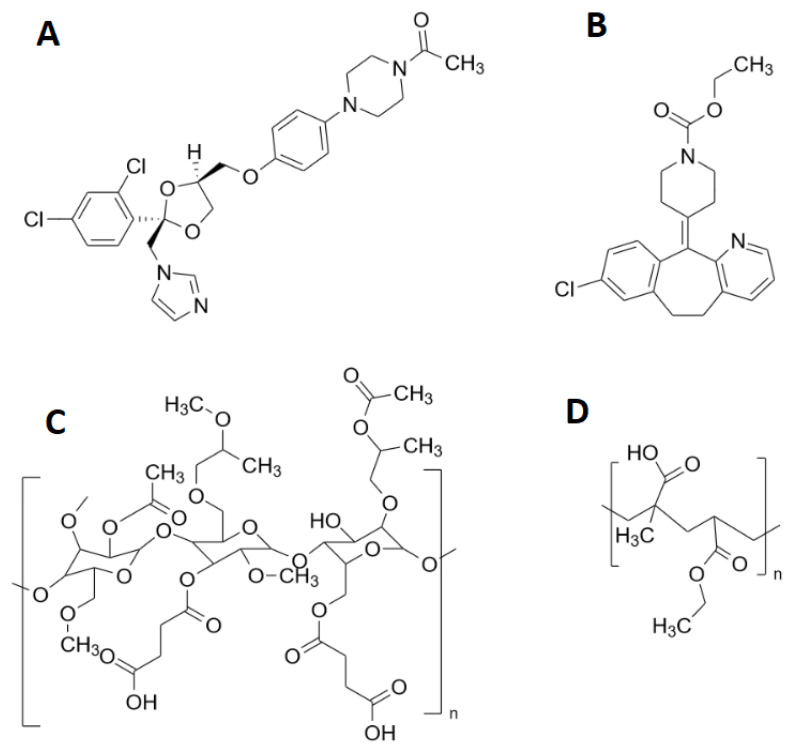
Chemical structures of the used materials: (**A**) ketoconazole; (**B**) loratadine; (**C**) Hydroxypropyl methylcellulose acetate succinate; and (**D**) Eudragit L100-55.

Ketoconazole (KCZ, Figure 1A) and Loratadine (LRD, Figure 1B) are weak bases and were used as model drugs, as both belong to BCS class II and are used as model drugs. Hydroxypropyl methylcellulose acetate succinate (HPMC-AS, Figure 1C) and Eudragit L100-55 (EL100-55, Figure 1D) were chosen as polymers, because both exhibit pH-dependent solubility in the intestinal tract, as the combination of weak bases with pH-dependent soluble polymers showed a significant improvement in bioavailability compared to non-pH-dependent soluble polymers [24,25,26].

## 2. Materials and Methods

### 2.1. Materials

The model drugs ketoconazole (KCZ) and loratadine (LRD) were purchased from Sris Pharmaceuticals (Hyderabad, India). HPMCAS LG (hydroxypropyl-methylcellulose acetate succinate, wt%: methoxyl 20–24%, hydroxypropyl 5–9%, succinyl 14–18%; Mw = 18,000, HPMC-AS) was donated from Shin-Etsu Chemical (Tokyo, Japan). Eudragit L100-55 (methacrylic acid ethylacrylate copolymer, ratio 1:1, Mw = 320,000, EL100-55) was donated by Evonik (Darmstadt, Germany). Cellets 1000 (microcrystalline cellulose starter pellets, 1000–1400 µm) were provided by Glatt Pharmaceutical Services (Binzen, Germany). A detailed list of the pellets’ characteristics is shown in Table 1. Ethanol 96% (*v*/*v*) (technical grade) used in the sample preparation, and methanol (analytical grade) used for the HPLC analytics as well as the buffer salts disodium mono-hydrogen phosphate dodecahydrate (Na_2_HPO_4_·12H_2_O) and monosodium dihydrogen phosphate dodecahydrate (NaH_2_PO_4_·12H_2_O) were obtained from VWR Chemicals GmbH (Darmstadt, Germany).

### 2.2. Methods

#### 2.2.1. Spray Drying (SD)

For spray drying (SD), a laboratory spray dryer B-290 equipped with an Inert Loop B-295 and a dehumidifier B-296 from Büchi Labortechnik GmbH (Essen, Germany) was used. To nebulize the spray solution, the spray dryer was equipped with a cooled two-fluid nozzle (Ø of 0.7 mm) with a 2.0 mm cap. The applied feed rate of solution as well as the nitrogen spray gas flow and the outlet temperature were kept constant at 5 g/min, 45 mm (corresponding to approximately 0.54 m^3^/h) and 30 ± 1.0 °C. The volume flow was kept constant at 25.0 ± 2.0 m^3^/h. To prepare the spraying solutions, the API and polymer were dissolved in ethanol 96% (*v*/*v*) under stirring (solid content of 10% (*w*/*w*)). Prior to spraying, each solution was sonicated for 15 min to ensure complete dissolution of the components. The prepared SD powder had a drug load of 20% (*w*/*w*) and the used compositions are listed in Table 2.

The prepared SD powders were dried for 24 h under vacuum conditions at 30 °C using a VD 53 vacuum oven (BINDER GmbH, Tuttlingen, Germany) to ensure an almost-complete removal of residual solvents. After drying the powder was compacted to simulate the downstream roller compaction via briquetting, using a pneumatic hydraulic tablet press (FlexiTab, Röltgen GmbH & Co. KG, Solingen, Germany) equipped with a 20 mm flat face tooling. The compaction force was set at 20 kN (corresponding to a compaction pressure of approximately 63.6 MPa) to obtain solid briquettes. Afterwards, the compacted samples were milled with a laboratory centrifugal mill equipped with a 2 mm sieve (ZM 1, Retsch GmbH, Haan, Germany). Subsequently, the ground materials were sieved with a sieve shaker (AS 200, Retsch GmbH, Haan, Germany) at an amplitude of 50% for 2 min to obtain three different sieve fractions (1000–2000 µm, 710–1000 µm; 500–710 µm).

#### 2.2.2. Pellet Coating (PC)

For pellet coating (PC) a laboratory scale fluid bed system Mini Glatt equipped with a Micro-Kit (Glatt GmbH, Binzen, Germany) was used. The coating was applied with a 0.5 mm two-fluid nozzle in bottom spray using the special bottom plate of the Micro-Kit to emulate a three-fluid nozzle with micro-climate. In the beginning, the machine was filled with 25.0 g of Cellets^®^ 1000. The following process parameters were maintained throughout the process: Process gas flow 30 m^3^/h, product temperature 30.0 ± 1.0 °C (resulting inlet temperature 32–35 °C), spray pressure 1.5 bar and spray rate 1.0 ± 0.2 g/min. The final pellets had a theoretical drug-load of 10% (*w*/*w*) due to the fact that ASD and core pellets were used in a 1:1-ratio. The used compositions are listed in Table 2 in more detail. To prepare the spraying solutions, the API and polymer were dissolved in ethanol 96% (*v*/*v*) under continuous stirring (solid content of 10% (*w*/*w*)). Prior to spraying, each solution was sonicated for 15 min to ensure complete dissolution of the components.

Subsequently, the coated pellets were manually sieved with a 2 mm mesh to eliminate multicore pellets. The pellets were dried under vacuum for 24 h at the same conditions as the SD powder.

#### 2.2.3. Gas Chromatography (GC)

The residual solvent content of ethanol after drying was determined using a Focus GC connected to a TriPlus SH Autosampler unit (Thermo Fischer Scientific, Waltham, MA, USA). The analysis was performed in headspace mode on an FS_CS_624 capillary column (length: 30 m, inner diameter of 0.32 mm, film diameter of 1.8 µm, 6% cyanopropylsiloxane, CS—Chromatographie Service GmbH, Langerwehe, Germany) and detected with a flame ionization detector at 240 °C. Samples of 30 mg were accurately weighed and mixed with 1.0 mL phosphate buffer system (PBS) at pH 6.8. After incubation at 80 °C for 10 min, 1.0 mL of the gas phase was injected into the GC. The column oven was heated from 60 to 80 °C at a rate of 2 °C/min and then heated to 150 °C at a rate of 10 °C/min. Synthetic air with a gas flow of 2.0 mL/min was used as carrier gas and nitrogen for a 1/5 spit flow during injection.

#### 2.2.4. High Performance Liquid Chromatography (HPLC) Analysis

An assay of SD-granules and SD-pellets was performed on a Shimadzu LC-2030 3D Plus HPLC system (Shimadzu Corp, Kyoto, Japan) equipped with a C18 reverse-phase column (Inertsil ODS-3, GL Sciences, Tokyo, Japan; 250 mm length, 4.6 mm inner diameter, and 5 µm particle size). For both API, the eluent consisted of acetonitrile and 10 mM phosphate buffer, pH 7.4 (70/30, %*v*/*v*) at a flow rate of 1.0 mL/min. The column temperature was set to 40 °C, the autosampler was kept at 25 °C, and injection volume was 10 µL. The detector was set to 220 nm for both KCZ and LRD. Linearity was confirmed from 5 to 50 µg/mL. Prior to analysis, the coatings of 100 mg of pellets were dissolved in 100 mL of methanol and filtered through PTFE syringe filters (pore size 20 µm).

#### 2.2.5. Dynamic Image Analysis

For determination of the particle size distribution, the calculation of two shape factors (aspect-ratio and sphericity) and the specific surface area (*S_m_*, [cm^2^/g]) within a dynamic image analysis a Camsizer X2 (Retsch GmbH, Haan, Germany) equipped with the gravimetric module X-Fall was used. The set particle size range was 10–4000 µm, and was divided into 50 size fractions. The particle width (*x_c min_*) was chosen as the comparative diameter, as this best reflects the particle distribution of a sieve used for particle separation. The determined shape factors were aspect-ratio (*b*/*l*) and sphericity (*SPHT*).

The *b*/*l* represents the general shape appearance and was determined as the width/length-ratio, which is determined from the particle width (*x_c min_*) and the particle length (*x_Fe max_*) (Equation (1)).

(1) $$\frac{b}{l}=\frac{x_{c\;min}}{x_{Fe\;max}},$$

The SPHT shows the roundness of the particles and was determined from the particle circumference U and the particle area A (Equation (2)).

(2) $$SPHT=\frac{4\pi *A}{U^{2}},$$

Additionally, the size span (*SPAN*) was determined from the median (*d*_50_) and the two outer percentiles (*d*_10_, *d*_90_), and used to represent the width of the particle size distribution (Equation (3)).

(3) $$SPAN=\frac{d_{90}-d_{10}}{d_{50}},$$

Using *S_m_*, the total outer particle surface area (*TOPS*) was calculated as the product of the sample mass used in the non-sink dissolution experiments *m_d_* and the *S_m_* (Equation (4)).

(4) $$TOPS\;\left[{\mathrm{cm}}^{2}\right]=S_{m}\;\left[\frac{{\mathrm{cm}}^{2}}{g}\right]*m_{d}\;\left[g\right],$$

#### 2.2.6. Pycnometric Density

The pycnometric density (PD) was obtained using a Belpycno L helium pycnometer (Microtrac MRB GmbH, Haan, Germany) equipped with a 40 cm^3^ sample chamber. For the measurements, an accurately weighed and preheated sample was placed in the chamber and the measuring cycles were repeated up to 20 times or until a standard deviation of 0.01% was reached. The density was used for the calculation of the specific surface area described in Section 2.2.5.

#### 2.2.7. Fourier-Transform Infrared Spectroscopy (FT-IR)

A Spectrum Two LiTa (Perkin Elmer LAS GmbH, Rodgau, Germany) equipped with an attenuated total reflection (ATR) accessory was used to investigate solid-state molecular interactions. For each dataset, six scans in a spectral range of 400–4000 cm^−1^ were recorded. All raw materials, physical mixtures and prepared ASDs were measured.

#### 2.2.8. Scanning Electron Microscopy (SEM)

For investigating the morphology of the various ASDs, a SU 3500 scanning electron microscope (Hitachi High Technologies, Krefeld, Germany) equipped with a scattering electron (SE) detector was used. SE images were acquired at a variable pressure mode of approximately 30 Pa and an accelerating voltage of 5 kV. The samples were mounted with conductive carbon patches and prior investigation sputtered with gold at 1.65 kV for 4 min under vacuum to reduce the electrostatic charge of the samples. In addition, the pellets were broken between two ceramic plates and the broken pellets were fixed on a sample holder to investigate a cross-sectional area of the ASD layer of the pellets.

#### 2.2.9. X-ray Powder Diffraction (XRPD)

The measurements were performed in reflection mode with an X’Pert MRD Pro (PANAnalytical, Almelo, The Netherlands) equipped with an X’Celerator detector and nickel filtered CuKα_1_ radiation (λ = 1.5406 Å) at 45 kV and 40 mA. The scanning range observed was between 5° and 45° 2θ with 0.017° 2θ measuring steps. The measuring step time was set to 0.5 s and the sample plate was placed in a sample spinner at 30 rpm. The samples were ground for 10 s at 20 Hz in a laboratory ball mill (Mixer Mill MM 400, Retsch GmbH, Haan, Germany) to obtain a fine powder before analysis.

#### 2.2.10. Differential Scanning Calorimetry (DSC)

The DSC measurements were carried out on a Mettler-Toledo DSC 2 (Mettler-Toledo GmbH, Gießen, Germany) equipped with a nitrogen cooling system. Samples of 10 mg were accurately weighed and placed in aluminum crucibles with pierced lids. For the measurement, a multi-frequency modulation (TOPEM-mode) with a background heating rate of 2 K/min from 0 to 165 °C was applied to determine the glass transition (Tg) temperature of the samples. A constant nitrogen flow of 30 mL/min was applied.

#### 2.2.11. Determination of Solubility

For determination of the equilibrium solubility (*c_Eq_*) of KCZ and LRD at pH 6.8, the shake flask method was performed for 48 h. Subsequently, 20.0 mg of API was dispersed in 50.0 mL of phosphate buffer (PBS) adjusted to pH 6.8 in a volumetric flask and stored in a tempered water bath (37 °C) for 48 h. Afterwards, the filtered samples were analyzed by HPLC (see Section 2.2.4). The *c_Eq_* was then used as a reference value to determine the *supersaturation factor*, as the quotient of the *c_Eq_* and *c_max_* of the respective ASD (Equation (5)).

(5) $$Supersaturation\;factor=\frac{c_{max}}{c_{Eq}},$$

#### 2.2.12. Non-Sink Dissolution

Since the polymers used are both pH-dependent soluble and enteric-coated, the dissolution of the samples was investigated under non-sink conditions at pH 6.8 (PBS) for 3 h. A Sotax AT7 with paddle (USP apparatus II; Sotax AG, Basel, Switzerland) was used for the dissolution studies. The paddle speed was set to 75 rpm and the media volume was 750.0 mL. The samples sizes relating to 150 mg of KCZ (equal to a theoretical maximum concentration of 0.2 mg/mL) and 30 mg LRD (equal to a theoretical maximum concentration of 0.04 mg/mL). The concentration over time of the dissolved APIs were measured online with a diode array UV/VIS spectrophotometer (Agilent 8453, Agilent Technologies GmbH, Waldbronn, Germany).

The dissolution rate was determined for the linear part of the dissolution curve before reaching *c_max_* using linear regression.

#### 2.2.13. Physical Stability

To investigate the physical stability of the ASDs with regard to recrystallisation, the ASD samples were filled into glass vials with punctured lids and stored for 6 months under various conditions. In accord with the WHO-Guideline [27], the storage conditions were 25 °C and 60% relative humidity (rH). In addition, the samples were also stored over desiccant for 6 months at 40 °C to verify thermal stability.

Since the API release and other quality properties of an amorphous solid dispersion can be considered as a function of its solid state [28], the analysis of the samples was limited to the solid state, by performing DSC and XRPD measurements.

In addition, the water uptake of the samples stored under 25 °C and 60% rH was investigated by using a Karl-Fischer-titrator (V30S, Mettler-Toledo GmbH, Gießen, Germany) equipped with a Autosampler oven (Stromboli, Mettler-Toledo GmbH, Gießen, Germany). A quantum of 200 mg of sample were weighed into a vial and heated at 80 °C. For the determination of the water content Methanol (HPLC-grade) and CombiTitrant 2 (containing Iodine, 2-Methylimidazole and Imidazole, Merck KGaA, Darmstadt, Germany) was used.

## 3. Results

### 3.1. Sample Preparation

The resulting process parameters, such as process time and process yield of the sample preparation, are shown in Table 3. In SD, about 55 g of granules (yield 55%) could be obtained from 100 g of material input (excluding solvents). In the case of PC, on the other hand, almost all of the 50 g of material input (excluding solvents) could be converted into coated pellets (yield: 99.67%). However, at 9.2 h for 100 g, PC is considerably slower than SD (4.1 h) under the investigated process parameters, which, on the other hand, was balanced by the limited yield of the SD process.

### 3.2. Sample Characterization

#### 3.2.1. Particle Characterization

The API content determined by HPLC is shown in Table 2. For all samples an assay of approx. 93–97% of the respective API content was obtained.

**Figure 2:**
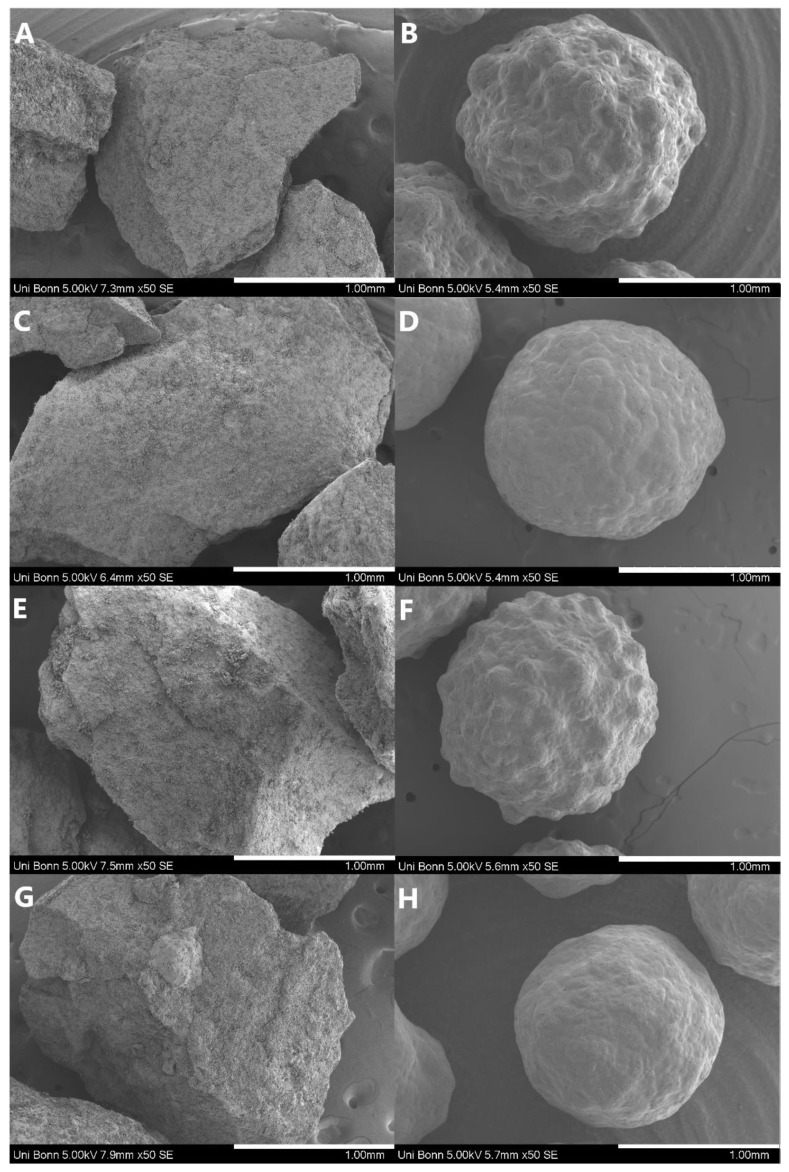
SEM-images of all prepared samples, containing: (**A**) KCZ_HPMC-AS_SD, (**B**) KCZ_HPMC-AS_PC, (**C**) KCZ_EL100-55_SD, (**D**) KCZ_EL100-55_PC, (**E**) LRD_HPMC-AS_SD, (**F**) LRD_HPMC-AS_PC, (**G**) LRD_EL100-55_SD, and (**H**) LRD_EL100-55-PC. All samples were sputtered with gold and observed with a Hitachi SU3500 at 5.0 kV in SE-mode and a 50-time magnification. The white scale bar represents 1 mm.

The additional results of the particle characterization are given in Table 4. The d_50_ of the prepared pellets were in the same range as the d_50_ of the largest sieve fraction of the milled SD particles. Considering that the raw pellets had a core diameter of appr. 1267.19 µm, the thickness of the layered ASD is 77.84 µm for KCZ_HPMC-AS_PC, 119.79 µm for KCZ_EL100-55_PC, 142.70 µm for LRD_HPMC-AS_PC and 147.64 µm for LRD_EL100-55_PC. The width of the PSD of the pellets was tighter (SPAN: 0.153–0.249; KCZ_EL100-55_PC and LRD_HPMC-AS_PC) than the particle size distribution of the sieve fractions of SD (SPAN: 0.460–1.019; LRD_EL100-55_SD_710-1000; KCZ_HPMC-AS_SD_500-710) which is attributed to the uniformity of the raw pellets indicated by the shape factors. The b/l-ratio of the PC was between 0.898 (LRD_HPMC-AS_PC) and 0.912 (KCZ_HPMC-AS_PC) which is also related to the uniform roundness of the starter cores. In contrast, the b/l-ratio of the milled SD sieve fractions varied between 0.627 (LRD_HPMC-AS_SD_500-710) and 0.673 (LRD_EL100-55_SD_500-710 or LRD_EL100-55_SD_1000-2000), which indicates a much more heterogeneous particle shape, one also shown in SEM-images (Figure 2). Therefore, for sphericity, the same difference was observed between SD particles (0.5–0.6) and PC pellets (0.8–0.9). In addition, a difference between the polymers used was identified for the PC samples. The pellets coated with HPMC-AS showed a SPHT of around 0.87 and the pellets coated with EL100-55 showed a SPHT around 0.94, which was likely attributable to the different surface texture of the pellets (Figure 2).

The measured densities of the pellets were always about 0.07 g/cm^3^ higher in particle density then the SD particles, which is attributed to both the higher pycnometer density of the starter pellets (1.452 g/cm^3^) and the respective volume ratio of starter pellet to coated ASD of about 2:1. The net density of the ASD layer coated onto the starter pellets is the same as that of the SD particles. In addition, the particle density of the samples containing HPMC-AS as polymer were usually 0.04 g/cm^3^ higher than the samples containing EL100-55.

The specific surface area (*S_m_*) of the pellets was always smaller than the specific surface area of the largest sieve fraction of the compacted SD particles. This is mainly due to the difference in density between the samples. In addition, due to their very similar density and their uniform shape, all pellets exhibited an almost identical specific surface area of about 40 cm^2^/g. For the compacted SD particles, *S_m_* increased strongly with decreasing *d*_50_, as expected.

For KCZ_HPMC-AS_PC, the determined residual solvent content was lowest at 2680 ± 220 ppm and highest for LRD_HPMC-AS_SD at 3520 ± 690 ppm, respectively. The measured residual solvent contents were all below the regulatory limit of 5000 ppm (equivalent to 0.5% (*w*/*w*)) [29]. No clear trend in between the manufacturing methods, the APIs or the polymers were observed.

**Figure 3:**
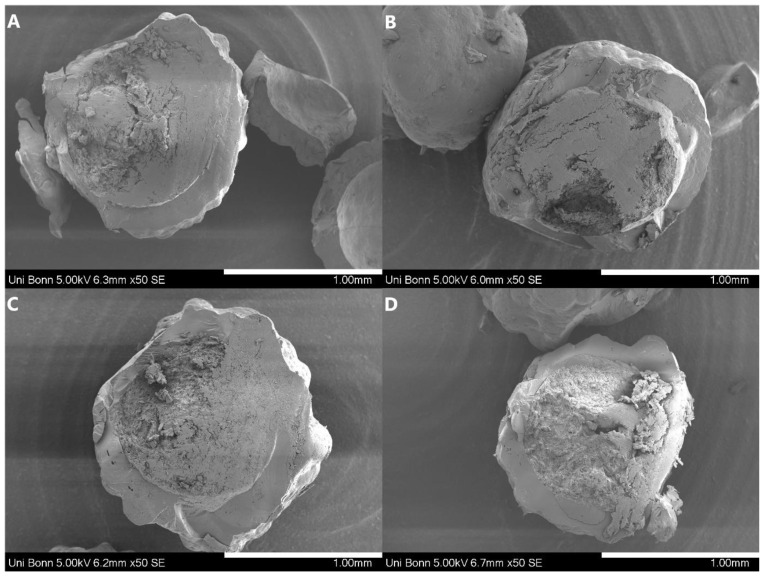
SEM-images of cross-sectional areas of the prepared pellets: ((**A**) KCZ_HPMC-AS_PC; (**B**) KCZ_EL100-55_PC; (**C**) LRD_HPMC-AS_PC; (**D**) LRD_EL100-55_PC). All samples were sputtered with gold and observed with a Hitachi SU3500 at 5.0 kV in SE-mode and a 50-time magnification. The white scale bar represents 1 mm.

The SEM images, which are shown in Figure 2, reveal the differences in surface structure of the different samples. The SD samples had an irregular shape with a rough and uneven surface structure where the compacted small particles of the spray drying are still visible. The ASD-pellets had, as expected, a much more spherical shape and smooth closed surface. The highly magnified surface images (Figure S1) exhibited the porosity of the SD particles in contrast to the pellets, as the individual SD particles, which had been compressed into granules, could still be seen in these images. The pellets containing HPMC-AS (Figure 2B,F) presented a rather uneven and “bubbly” surface structure. The cross-sectional area of the PC particles, however, revealed themselves to be completely solid, and without any gas entrapment (Figure 3), which indicates that the structural differences between the pellets were not caused by air inclusions, but were determined by the respective polymer properties.

#### 3.2.2. Solid State Characterization

For the unprocessed APIs, complete crystallinity was proved by their observed melting points in DSC (KCZ: 151.33 ± 0.49 °C, LRD: 138.40 ± 0.99 °C). The Tgs of the unprocessed polymers were found at 119.34 ± 0.89 °C for HPMC-AS and at 122.56 ± 0.16 °C for EL100-55. The thermograms are presented in Supplementary Data (Figure S2). The Tg of the samples prepared (see Table 4) with HPMC-AS as polymer, regardless of the preparation method, ranged from 89.86 °C (LRD_HPMC-AS_SD) to 91.92 °C (KCZ_HPMC-AS_PC). For the samples prepared with EL100-55, the glass transition temperatures ranged from 108.74 °C (LRD_EL100-55_PC) to 113.00 °C (LRD_EL100-55_SD).

**Figure 4:**
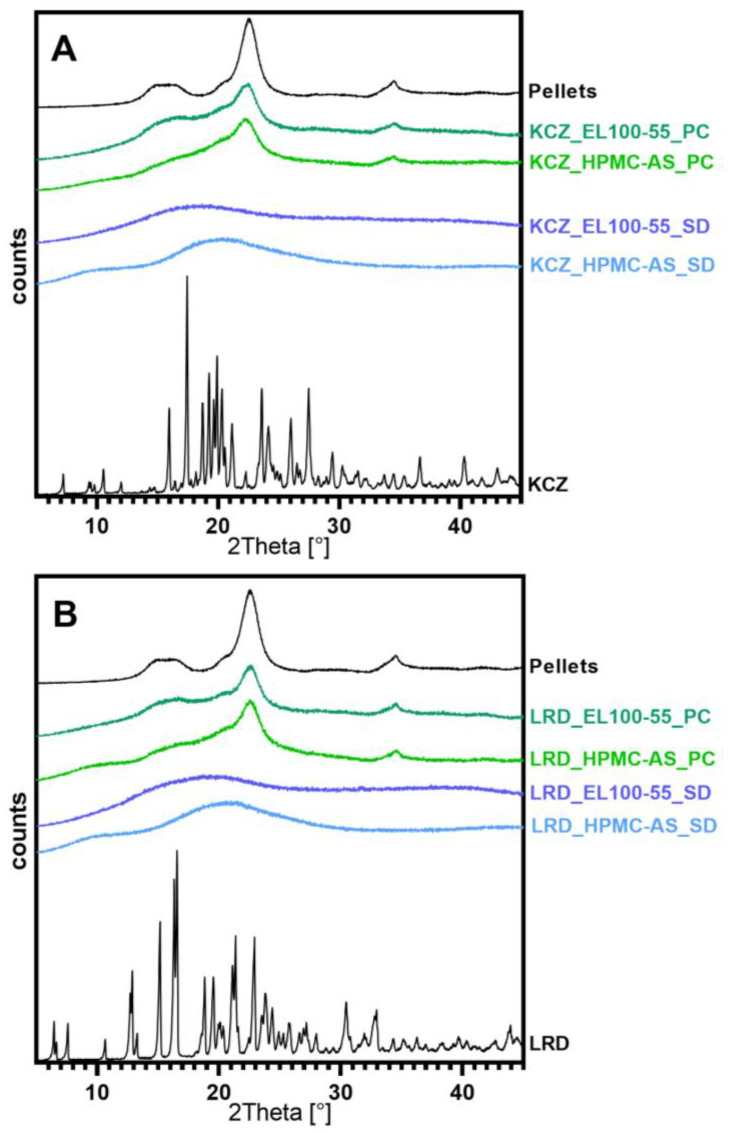
XRD-results of all prepared samples and pure API and pure Pellets (black lines), (**A**) Ketoconazole and (**B**) Loratadine (light blue line: HPMC-AS_SD; dark blue line: EL100-55_SD; light green line: HPMC-AS_PC; dark green line: EL100-55_PC).

Figure 4 shows the XRD spectra of all samples, together with the respective pure API and uncoated starter pellets. A representation of the XRD spectra of all individual excipients can be found in the Supplementary Data (Figure S3). KCZ showed several major reflection peaks at 15.96°, 17.48°, 18.75°, 19.26°, 19.98°, 20.34°, 23.60°, 26.06° and 27.50° 2Theta(2θ). In addition, minor reflexes were observed at 7.25°, 10.58°, 21.28°, 24.17°, 29.53°, 36.76° and 40.30° 2θ (Figure 4A). LRD exhibited several major reflection peaks at 12.75° (double), 15.14°, 15.51° (double), 18.82°, 19.62°, 21.21° (double) and 22.95° 2θ. In addition, minor reflexes at 6.53°, 7.69°, 10.73°, 13.26°, 23.89°, 24.39°, 25.84°, 30.39° and 32.78° 2θ were observed (Figure 4B). None of the produced samples showed any of the reflexes corresponding to KCZ or LRD, respectively. Only the halo reflection pattern resulting from the polymers used and the reflexes of the starter pellets used were visible in the spectra. This indicates a complete amorphization of the APIs and the lack of residual crystallinity in the samples.

**Figure 5:**
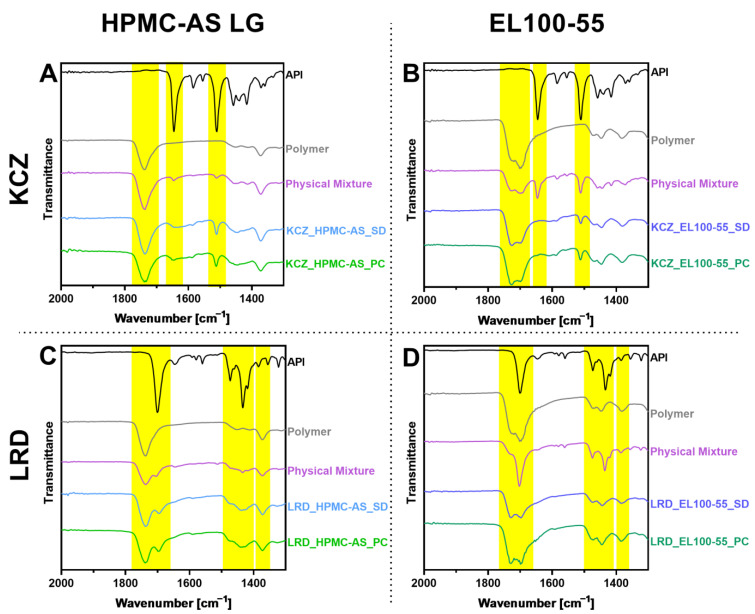
FT-IR-measurements of all prepared samples, pure APIs, pure polymers and their respective physical mixtures containing (**A**) KCZ and HPMC-AS L; (**B**) KCZ and EL100-55; (**C**) LRD and HPMC-AS; and (**D**) LRD and EL100-55. Yellow bars representing region of interest. Measurements were performed with a FT-IR Spectrum two LiTa (Perkin Elmar). 1300–2000 cm^−1^.

The FT-IR measurements (Figure 5) include the spectra of the pure APIs (black), the pure polymers (gray), their physical mixture (purple), the SD particles (blue) and spectra of the ASD pellets (green). Since most polymer/API interactions involve acid or amide groups, the region of interest for the evaluation was limited to the range between 1300–2000 cm^−1^ [30]. The evaluation of the interactions was focused on the yellow highlighted ranges, since these represent particularly characteristic peaks of the polymers and/or the APIs.

For the polymer HPMC-AS, at 1737.1 cm^−1^ a broad band of the stretching of the carbonyl moiety was clearly identified.

For EL100-55, at 1727.7 cm^−1^ and 1698.6 cm^−1^, the characteristic double band of EL100-55 presented itself with a slightly higher intensity at 1698.6, which could be assigned to the ethanolic acids and the ethanolic acid esters.

KCZ showed a strong CO signal at 1645.3 cm^−1^, from the carboxyl group. Several signals originated from the aromatic carbons (1584.5; 1554.5 and 1510.5 cm^−1^), with the signal at 1510.5 cm^−1^ being the most intense.

The LRD spectrum showed a very pronounced band of the carboxy group at 1700.5 cm^−1^. At a wavenumber of 1472.9 cm^−1^, the vibration of the carbamide moiety was recognizable and at 1433.6 cm^−1^ the vibrations of the aromatic carbons were in evidence.

In the measured physical mixtures, the spectra were linear combinations of the respective individual spectra, and the dominant bands were present in each case (yellow stripes).

For the ASD samples examined, no differences in the IR-spectra among the various production methods could be observed. Therefore, the following paragraph does not distinguish the ASD origin.

For the combination of KCZ and HPMC-AS (Figure 5A), the CO band of the polymer was retained, but the CO peak of the API disappeared.

In the combination of KCZ and EL100-55 (Figure 5B), the CO band of KCZ disappeared, but the CN band remained visible. In addition, a reversal in the intensities of the two bands could be seen with the CO double band of the EL100-55.

For the combination of LRD and HPMC-AS (Figure 5C), the changes previously described for KCZ could not be detected. The spectra of the samples showed exactly the same peaks at the same wavenumbers as the spectrum of the physical mixture.

However, as for the combination of LRD and EL100-55 (Figure 5D), the CO band of the LRD, which was clearly recognizable in the spectrum of the PM, disappeared in the double band of the EL100-55 after manufacturing. Within this double peak, a weak intensity reversal as described above also occurred. In addition, the signals that were clearly visible at 1400 decreased pronouncedly.

### 3.3. Non-Sink Dissolution Testing

**Figure 6:**
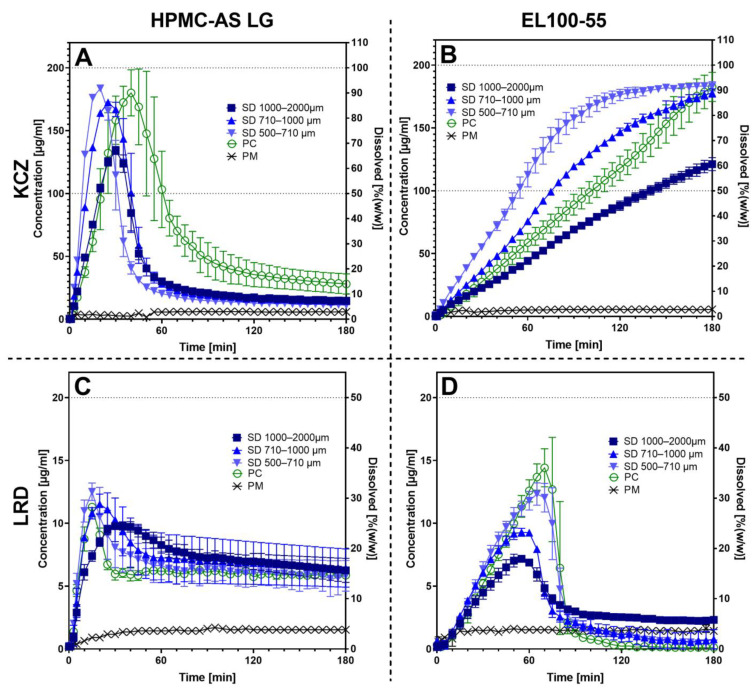
Dissolution profiles of all prepared samples containing (**A**) KCZ and HPMC-AS L; (**B**) KCZ and EL100-55; (**C**) LRD and HPMC-AS; and (**D**) LRD and EL100-55. Coated pellets are represented by green open circles (○). The different sieve fractions of the SD are represented by blue filled symbols, with squares for 1000–2000 µm (■), triangles for 710–1000 µm (▲), and turned triangles for 500–710 µm (▼). The physical mixtures are presented by black crosses (×). The dissolution was observed at pH 6.8 in PBS-buffer over a period of 180 min.

The non-sink dissolution studies are shown in Figure 6. In addition, release-specific metrics are presented in Supplementary Data (Table S1). In the shake-flask experiments, an equilibrium solubility at pH 6.8 of 5.13 ± 0.13 µg/mL was determined for KCZ and 1.05 ± 0.05 µg/mL for LRD. In all release tests, a supersaturation factor of 4.8 up to 30.7 compared to the PM was achieved with all tested ASD formulations.

For the combination KCZ and HPMC-AS (Figure 6A), each sieve fraction released their maximum amount of KCZ within the first 40 min (*t_max_*: 20.0–41.7 min). After reaching the *c_max_*, a large amount of the KCZ precipitated within an hour, resulting in a final concentration after the observed experiment time (*c_end_*) that was slightly higher than the end concentration of the PM (*c_end_*: 14.9–36.7 µg/mL). For the smallest sieve fraction of the SD (KCZ_HPMC-AS_SD_500-710) the highest supersaturation was observed with a factor of 35.8. As the particle size increased, the supersaturation factor of the SD sieve fractions decreased steadily (33.6 for KCZ_HPMC-AS_SD_710-1000 and 26.2 for KCZ_HPMC-AS_SD_1000-2000). The pellets (KCZ_HPMC-AS_PC) reached a supersaturation factor of 35.5. No supersaturation was achieved for the PM. After the final concentration of 5.88 µg/mL (2.94% (*w*/*w*)) was reached, the concentration remained constant over the remaining observation period.

In the case of the combination KCZ and EL100-55 (Figure 6B), the release kinetics changed completely compared to the formulations with HPMC-AS. KCZ concentration increased steadily over the entire test period to a final maximum, without precipitation (*c_ma_*_x_: 121.20–184.03 µg/mL and *t_max_* at 180.0 min). Comparable to the results observed for the combination of KCZ and HPMC-AS, the lowest supersaturation was achieved with the highest sieve fraction of SD particles (KCZ_EL100-55_SD_1000-2000), with a factor of 23.6. Again, the highest supersaturation factor was observed for the smallest sieve fraction of SD (KCZ_EL100-55_SD_500-710) at 30.7. Significantly higher supersaturation was found for the remaining sieve fractions of SD, as well as for the pellets. These formulations did not differ significantly in their supersaturation factors (34.7–35.9), but did differ in the dissolution rate observed within the first 10 min. The pellets showed the slowest and the smallest sieve fraction of the SD particles the fastest drug release (1.50 g/mL/min for KCZ_EL100-55_PC and 2.64 µg/mL/min for KCZ_EL100-55_SD_500-710). In addition, no supersaturation for the PM of KCZ and EL100-55 was observed, and *c_end_* of 5.60 µg/mL was reached.

The combination LRD and HPMC-AS (Figure 6C) showed a rapid release (*t_max_*: 15–33.3 min and *c_max_*: 9.90–12.51 µg/mL) with subsequent precipitation of LRD (*c_end_:* 2.75–6.44 µg/mL). The highest supersaturation factor (11.9) was observed for the smallest sieve fraction of SD (LRD_HPMC-AS_SD_500-710). The lowest supersaturation factor of 9.4 was observed for the largest sieve fraction of SD (LRD_HPMC-AS_SD_1000-2000). The pellets (LRD_HPMC-AS_PC) presented a supersaturation factor of 10.7. The PM reached a final concentration of 1.53 µg/mL and no supersaturation was noticeable.

For the last combination of LRD and EL100-55 (Figure 6D) all tested formulations showed dissolution for about one hour (*t_max_*: 53.3–71.7 min). The pellets (LRD_EL100-55_PC) reached the highest supersaturation factor of 13.9 with a *c_max_* of 14.62 ± 1.51 µg/mL. The lowest supersaturation factor (6.9) was observed for LRD_EL100-55_SD_1000-2000. After reaching a maximum, the LRD concentration dropped within 30 min, showing precipitation. LRD_EL_SD_1000-2000 dropped to a *c_end_* of 2.32 ± 0.07 µg/mL. The LRD concentration for all other formulations dropped to end concentrations between 0.77 ± 0.63 µg/mL for LRD_EL_SD_710-1000 and 0.12 µg/mL for LRD_EL100-55_PC, which was below the end concentration of the PM (*c_end_* = 1.44 ± 0.01 µg/mL).

### 3.4. Stability

The results of the stability investigation, performed for 6 months at 25 °C and 60% rH or 40 °C over desiccant, are shown in the Supplementary Data (Table S2: glass transition and water uptake; Figure S4: XRD measurements). The results showed no shift in Tg and no additional Tg steps that would indicate a phase separation compared to the initial measurements directly after production. Also, the XRD measurements showed no change in the diffractograms for both storage conditions as at the beginning of the investigation. The water uptake of the samples openly stored at 25 °C and 60% rH after 6 months ranged from 0.9 to 1.5% compared to its initial values (2.5–3.0%). It can be deduced that all samples are physically stable under the storage conditions investigated (25 °C/60% rH open and 40 °C/75% rH closed with desiccant), and showed no recrystallization tendency at all.

## 4. Discussion

### 4.1. Process

When evaluating the two manufacturing processes, the significant difference in the yield of the two processes is most prominent. Even so, in our SD experiments an average yield of only about 55% could be achieved. Low yield seems to be a common issue related to lab scale SD, resulting in yields between 50% and 70% without process optimization [31,32,33,34,35]. Even with process optimization the authors could achieve yields between 65% and 85% and only in one instance was a yield above 90% achieved, demonstrating a potential higher effort for process optimization in combination with SD. In contrast, with a yield of almost 100%, the finding is well above expectations for PC. In development, yields above 95% are mostly considered acceptable for fluid bed processes. Especially in early development phases, with very expensive and limited APIs [36], this process would have a clear benefit. However, this very good yield is achieved at the expense of process time. During a PC scale-up, process time increase by a factor of 2–6 dependent on the sizes and mass ratios in the larger systems [37].

With the current process design, it is possible to produce about 50 g of sample material in under 5 h with both processes, which would be sufficient for an early formulation development phase. However, for a possible scale-up of SD or PC, process optimization should be carried out for both processes.

To improve the yields in SD, the drying time of the droplets would have to be shortened, which can be achieved mainly by reducing the spray rate, which leads to smaller droplet sizes at the same spray pressure. However, this would also increase the process time by the same factor as the spray rate is reduced. Alternatively, a longer spray chamber could be employed, which would lead to an increased drying period.

Process optimization in the PC area would have to revolve primarily around seeking a reduction in the process time. This could be achieved by optimizing (increasing) the spray rate and the inlet temperature.

Since process optimization with the aim of maximizing yield in SD and PC always depends on several influencing factors at the same time, factors which can also influence each other, this is a point that requires further research and should be investigated, for example, by means of an appropriate design of experiments (DOE).

### 4.2. Sample Characteristics

The differences in particle morphology (size distribution, *SPAN*, shape factors and SEM images) are mostly related to the respective production methods, especially regarding the differences between SD and PC particles. However, within the PC formulations clear differences in surface morphology resulted in a significant difference of the shape factors (*SPHT* and *b*/*l*). The uneven, “bubbly” surface structure resulting in lower *SPHT* and *b*/*l* values observed with ASD-pellets containing HPMC-AS might be related to lower polymer/solvent interactions of HPMC-AS/ethanol solutions compared to, e.g., acetone/water mixtures [38]. In short, the cohesion between the polymer chains could be more pronounced and feasible to form aggregates during the sol-gel solid transformation. Obviously, this effect did neither result in a higher inner porosity (see Figure 3) nor the molecular dispersion of the drugs in HPMC-AS (Figure 4).

### 4.3. Solid State

Since the XRD diffractograms of all samples showed only the signals of the amorphous components and no residual crystallinity of the APIs (Figure 4), while the DSC did not show any melt endotherm from remaining API crystals, complete amorphization with all formulations and processes could be achieved. The absence of a second glass transition furthermore confirmed a single-phased ASD, while the detected Tg was only dependent on the formulation (API/polymer mixture), not the applied process. Despite the slower drying kinetics of the fluid bed process, the active ingredients had no tendency to recrystallize. As Alhalaweh et al. classifies both APIs as slow crystallizers, these results must be limited, as rapid crystallizers like phenytoin may not be fully converted successfully to the amorphous state with PC [39].

### 4.4. Non-Sink Dissolution

In general, the dissolution experiments for the ASDs containing KCZ as API and HPMC-AS or EL100-55 as polymer produced by PC or SD shown in this work confirmed previous results of our working group where the ASDs were produced by hot melt extrusion [40], proving a process-independent (PC, SD or HME) dissolution for KCZ and HPMC-AS or EL100-55.

**Figure 7:**
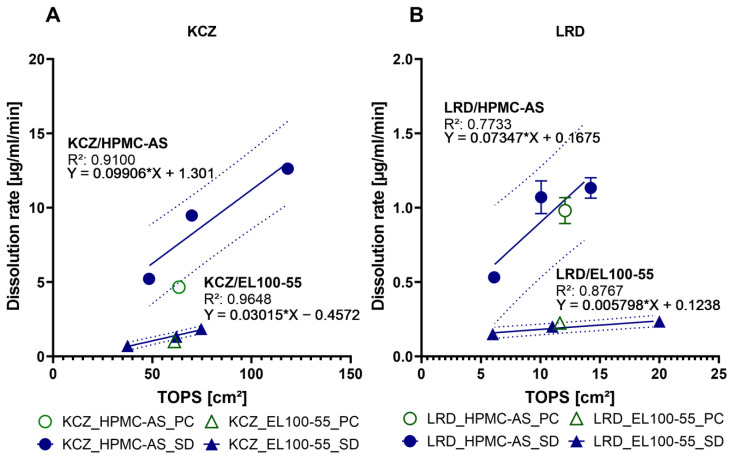
The linear relationship of the dissolution rate in [µg/mL/min] and the TOPS in [cm^2^] of prepared ASDs, containing: (**A**) KCZ and (**B**) LRD. The pellets represented by the hollow symbols and the SD particle represented by the filled symbols.

As expected, the dissolution rate increased with the specific surfaces of the various formulations available for the dissolution medium and an even linear relationship between the total outer surface area (TOPS) and the dissolution rate can be observed (Figure 7). Compared to the specific surface values, the TOPS values reflect the absolute surface value of the dissolution sample of the ASD pellet, eliminating the effect of the higher density of the neutral starter pellets within the PC formulations. With that, the accessible surfaces for the dissolution process for ASD-pellets and SD-particles are comparable. For three out of four cases, the dissolution rates of the pellets were almost identical with the dissolution rate of the medium sieve fractions (710–1000 µm) of the SD granules. Only for KCZ/HPMC-AS is the dissolution rate of the pellets close to the largest sieve fraction (1000–2000 µm) of the corresponding SD granules. Despite the small number of data points, it can be seen that in all cases, the dissolution rate of the pellets was within the 95% confidence interval of the respective SD granules (Figure 7). Therefore, it could be assumed that the rate-determining factor in initial drug release from these ASDs is their outer surface area and that the process is only slightly influenced by the inner particle structure. This would also be consistent with the Noyes-Whitney equation, which directly relates diffusion surface area to dissolution rate [41]. Despite the relationship deviates, in the case of KCZ_HPMC-AS_PC to 49.91%, from the linear relationship, a clear trend is noticeable. The reasons might be related to the low number of data points and the fact that *S_m_* was mathematically determined by PSD and is therefore based only on a mathematical model, which assumes that the particles are ideal spheres that could underestimate the actual particle shape, especially in combination with the uneven surface of HPMC-AS containing pellets or irregular shaped SD particles.

Although increased porosity was observed in the SEM for the SD particles (Figure S1 supplement), this did not appear to have a major impact on dissolution kinetics of the SD-granules. This could be related to the fact that the HPMC-AS and EL100-55 used first swell upon dissolution and form a thin phase-separated gel layer around the particle before going into solution [42,43]. Especially for EL100-55, other studies showed that a sustained release behavior occurs when used as an ASD polymer [40,44,45]. This could be caused by an increased interaction of the amine function of the APIs with the carboxylic acid moiety of the polymer. Borodkin and Yunker have already described the increased interactions between these two moieties [46].

The established relationship between outer surface (TOPS) and release rate should also be valid for pellets, i.e., a higher release rate should be achieved using smaller pellet cores. With that, an easy transfer from an established SD process to a PC process with comparable performance, but easier processability including less downstream process steps, would be possible.

## 5. Conclusions

In this study, we successfully prepared binary single phase ASDs of KCZ and LRD as weakly basic, slow crystalizing model APIs (drug load 20% (*w*/*w*)) using HPMC-AS or EL100-55 as pH-dependent soluble polymers via fluid bed pellet coating and spray drying. While the received ASD-pellets would not require further downstream processing other than capsule filling or tableting, the fine SD powder had to be transformed into dry granules. In combination with the slow crystallizers, KTZ and LOR, both manufacturing processes resulted in single-phased ASDs of high physical stability (up to 6 months) and similar dissolution performance when normalized to the total outer surface. The dissolution rate depends mainly on this total outer particle surface of the respective sample, independent of the manufacturing process, while the porosity of the sample had a minor impact on its dissolution behavior.

Especially for early formulation development, the high yield and ease of handling due to the pellet properties are strong advantages over the standard spray drying process. Nevertheless, the long process time in larger scale requires further process optimization in fluidized bed processing.

## Figures and Tables

**Table 1:** Pellet properties of Cellets 1000.

Pellet Properties	
d50 (xc min) [µm]	1123.44 (±7.36)
SPAN	0.166 (±0.002)
b/l	0.893 (±0.000)
SPHT	0.956 (±0.001)
Particle density [g/cm^3^]	1.452 (±0.016)
*S_m_* [cm^2^/g]	36.41 (±0.29)

d50: mean particle diameter determined by the particle width; SPAN: width of the particle distribution; b/l: aspect ratio; SPHT: sphericity; *S_m_*: specific surface area.

**Table 2:** Composition of the formulations used production techniques and the API-Yield of the acquired samples (*n* = 3).

Sample ID	API	Polymer	Comp. API/Polymer	Production Technique	Batch Size [g] (Solids)	Drug-Load Achieved [% (*w*/*w*)]	Assay [% (*w*/*w*)]
KCZ_HPMC-AS_SD	KCZ	HPMC-AS LG	20/80	SD	100	19.12 ± 0.37	96.97 ± 3.11
KCZ_HPMC-AS_PC	KCZ	HPMC-AS LG	20/80	PC	50	9.32 ± 0.50	93.18 ± 5.52
KCZ_EL100-55_SD	KCZ	Eudragit L100-55	20/80	SD	100	19.13 ± 0.11	95.72 ± 3.56
KCZ_EL100-55_PC	KCZ	Eudragit L100-55	20/80	PC	50	9.34 ± 0.55	93.44 ± 5.04
LRD_HPMC-AS_SD	LRD	HPMC-AS LG	20/80	SD	100	19.06 ± 0.73	95.30 ± 3.63
LRD_HPMC-AS_PC	LRD	HPMC-AS LG	20/80	PC	50	9.56 ± 0.28	95.56 ± 2.81
LRD_EL100-55_SD	LRD	Eudragit L100-55	20/80	SD	100	19.42 ± 0.72	97.09 ± 3.88
LRD_EL100-55_PC	LRD	Eudragit L100-55	20/80	PC	50	9.01 ± 0.45	92.43 ± 4.36

**Table 3:** The resulting process characteristics of the SD and PC processes. The mean value is shown with the standard deviation.

Preparation Technique	Average Process Time [h/100 g]	Average Process Yield [% (*w*/*w*)]
Spray drying	4.1 ± 0.2	54.45 ± 2.41
Pellet coating	9.2 ± 0.2	99.67 ± 1.16

**Table 4:** Results with standard deviation of the particle analysis, density measurement, GC and DSC.

Sample ID	Sieve Fraction [µm]	*d*_50_ [µm]	* SPAN *	*l*/*b*-Ratio	* SPHT *	* PD * [g/cm^3^]	* S_m_* [cm^2^/g]	Residual Solvent [ppm]	Tg [°C]
KCZ_HPMC-AS_SD	1000–2000	1491.1 ± 2.4	0.766 ± 0.003	0.644 ± 0.001	0.743 ± 0.004	1.287 ± 0.022	64.46 ± 0.83	3100 ± 790	91.92 ± 1.20 *
	710–1000	870.9 ± 0.9	0.618 ± 0.005	0.643 ± 0.001	0.744 ± 0.004		93.19 ± 1.05		
	500–710	605.4 ± 1.2	1.019 ± 0.010	0.641 ± 0.001	0.721 ± 0.005		157.68 ± 1.48		
KCZ_HPMC-AS_PC	-	1422.9 ± 1.5	0.193 ± 0.003	0.909 ± 0.000	0.877 ± 0.000	1.357 ± 0.014	42.27 ± 0.35	2680 ± 220	91.86 ± 0.48 *
KCZ_EL100-55_SD	1000–2000	1515.6 ± 1.0	0.639 ± 0.001	0.660 ± 0.000	0.774 ± 0.003	1.249 ± 0.031	50.02 ± 0.49	3220 ± 880	110.13 ± 0.99 *
	710–1000	882.4 ± 0.9	0.487 ± 0.006	0.665 ± 0.001	0.781 ± 0.003		82.73 ± 0.93		
	500–710	626.4 ± 1.1	0.488 ± 0.002	0.663 ± 0.001	0.782 ± 0.003		99.55 ± 1.54		
KCZ_EL100-55_PC	-	1506.8 ± 1.3	0.153 ± 0.002	0.912 ± 0.000	0.945 ± 0.000	1.312 ± 0.037	40.74 ± 0.86	3220 ± 400	109.72 ± 0.82 *
LRD_HPMC-AS_SD	1000–2000	1508.8 ± 2.6	0.820 ± 0.008	0.631 ± 0.001	0.741 ± 0.006	1.267 ± 0.047	40.73 ± 0.38	3520 ± 690	89.86 ± 2.47 *
	710–1000	905.2 ± 3.2	0.612 ± 0.005	0.631 ± 0.000	0.754 ± 0.005		64.07 ± 1.84		
	500–710	641.2 ± 1.5	0.620 ± 0.010	0.627 ± 0.001	0.755 ± 0.005		94.84 ± 1.43		
LRD_HPMC-AS_PC	-	1552.6 ± 1.2	0.249 ± 0.005	0.898 ± 0.000	0.867 ± 0.000	1.332 ± 0.016	40.23 ± 0.94	3240 ± 660	91.78 ± 2.33 *
LRD_EL100-55_SD	1000–2000	1510.1 ± 1.8	0.635 ± 0.003	0.673 ± 0.002	0.772 ± 0.002	1.228 ± 0.037	44.95 ± 0.68	3210 ± 330	113.00 ± 0.79 *
	710–1000	884.4 ± 1.6	0.460 ± 0.004	0.671 ± 0.000	0.772 ± 0.003		74.74 ± 0.43		
	500–710	610.0 ± 2.3	0.541 ± 0.001	0.673 ± 0.001	0.781 ± 0.004		139.60 ± 1.59		
LRD_EL100-55_PC	-	1562.5 ± 1.6	0.188 ± 0.007	0.898 ± 0.000	0.942 ± 0.000	1.307 ± 0.034	38.80 ± 0.34	3380 ± 290	112.08 ± 3.59 *

* For all combinations of API and polymer, no significant difference between the manufacturing methods was proved by one-way ANOVA analysis (α: 0.05).

## Data Availability

Not applicable.

## Supplementary Materials

The following supporting information can be downloaded at: https://www.mdpi.com/article/10.3390/pharmaceutics15030764/s1, Figure S1: SEM-images of all prepared samples containing (A) KCZ_HPMC-AS_SD, (B) KCZ_HPMC-AS_PC, (C) KCZ_EL100-55_SD, (D) KCZ_EL100-55_PC, (E) LRD_HPMC-AS_SD, (F) LRD_HPMC-AS_PC, (G) LRD_EL100-55_SD and (H) LRD_EL100-55-PC; Figure S2: Thermograms of DSC analysis of (A) KCZ and LRD (B) HPMC-AS and EL100-55, (C) thermograms of all prepared samples. All measurements were conducted in TOPEM Mode between 0° and 160 °C with a heat rate of 2 K/min; Figure S3: XRD-measurements of the used plain polymers; Figure S4: XRD-results of all prepared samples after storage for 6 months under different conditions (40 °C/0% rH and 25 °C/60% rH); Table S1: Results of the dissolution testing of all formulations showing c_max_, t_max_, AUC and dissolution rate determined by the linear fit prior c_max_; Table S2: Stability data: DSC-data and Karl Fischer after six months of storage under 25 °C and 60% rH or 40 °C and 0% rH.
