# Supplementary material for: Amorphous Solid Dispersions Layered onto Pellets—An Alternative to Spray Drying?
Source: Pharmaceutics. 2023 Feb 24;15(3):764. doi: 10.3390/pharmaceutics15030764 (PMC10054131; doi:10.3390/pharmaceutics15030764)
Supplement: Supplementary file 1 [file pharmaceutics-15-00764-s001.zip › pharmaceutics-2222189-supplementary.pdf]

pharmaceutics

MDPI

Supplementary Materials

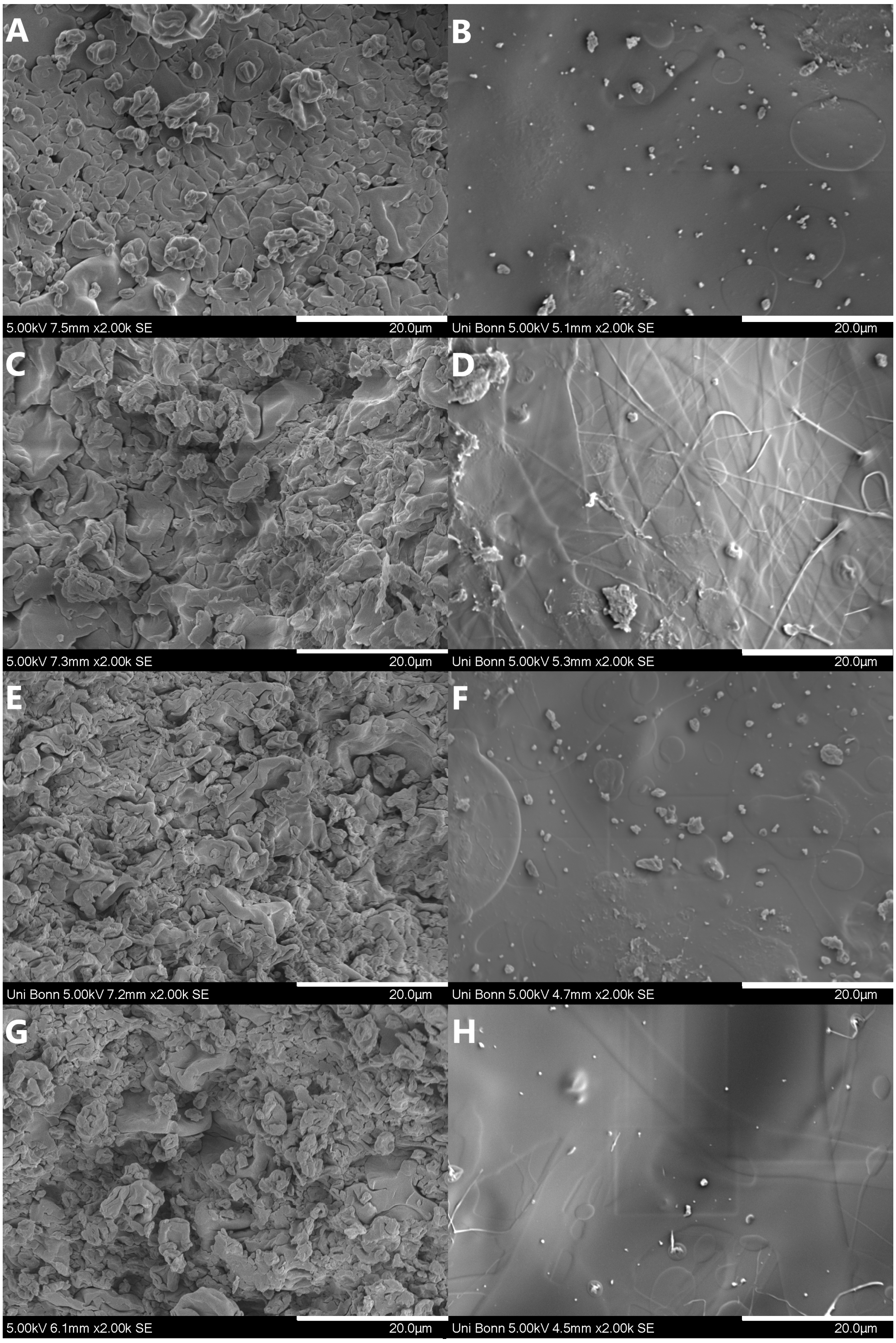

Figure S1. SEM-images of all prepared samples containing (A) KCZ_HPMC-AS_SD, (B) KCZ_HPMC-AS_PC, (C) KCZ_EL100-55_SD, (D) KCZ_EL100-55_PC, (E) LRD_HPMC-AS_SD, (F) LRD_HPMC-AS_PC, (G) LRD_EL100-55_SD and (H) LRD_EL100-55-PC. All samples were sputtered with gold and observed with a Hitachi SU3500 at 5.0 kV in SE-mode and a 2000-time magnification. The white scale bar represents 20 micrometers.

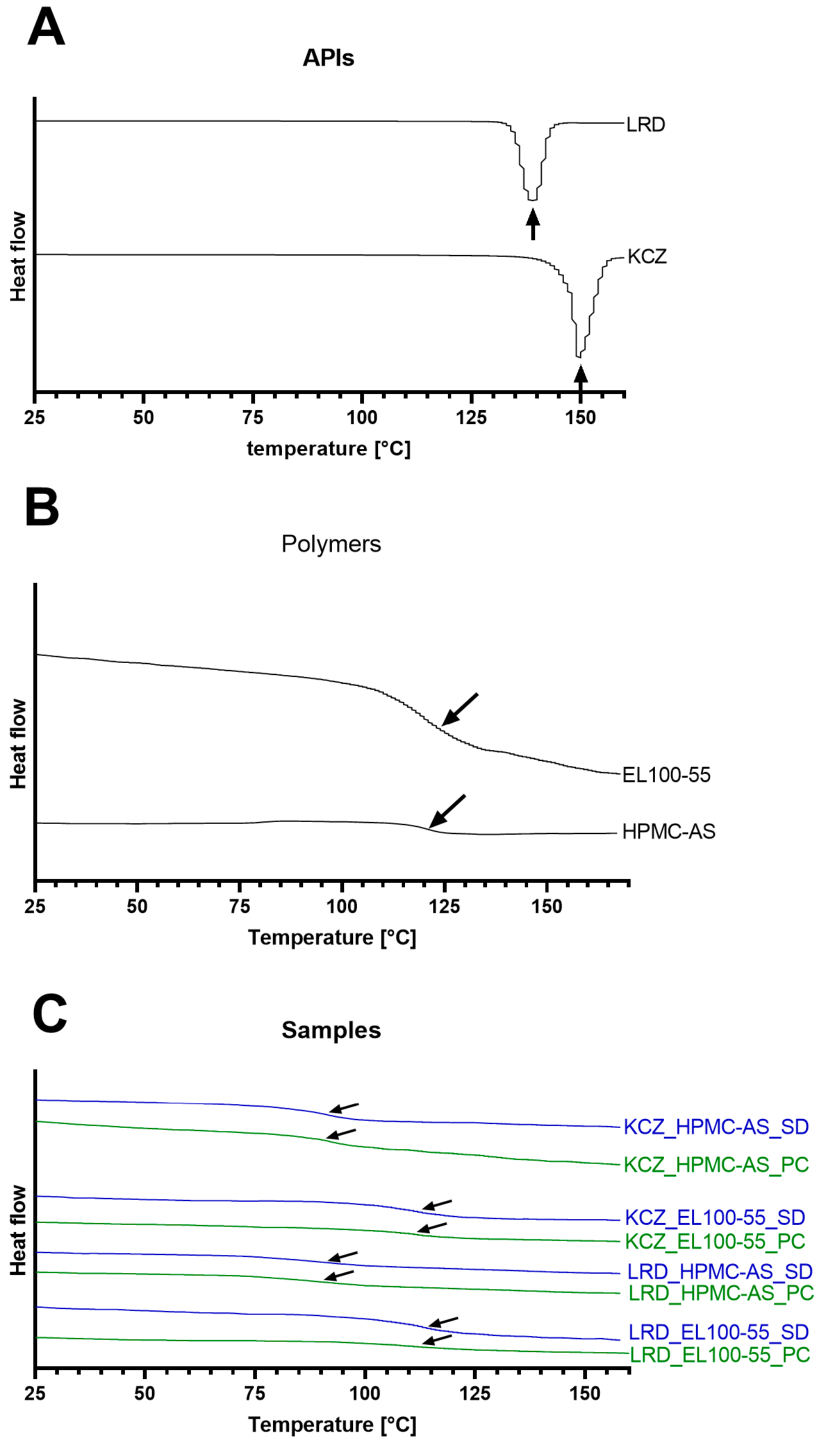

Figure S2. Thermograms of DSC analysis of (A) KCZ and LRD (B) HPMC-AS and EL100-55, (C) thermograms of all prepared samples. All measurements were conducted in TOPEM Mode between 0° and 160 °C with a heat rate of 2 K/min. The black arrow represents the T_M for A und the Tg for B & C.

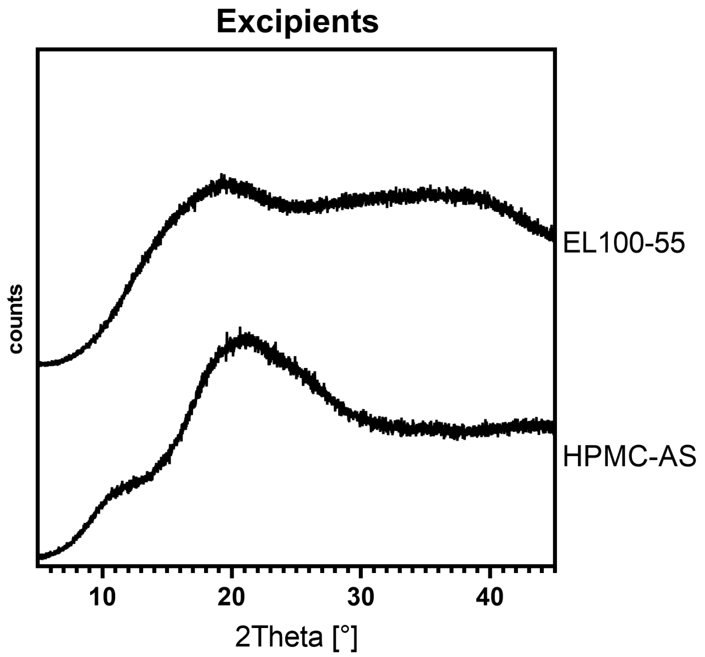

Figure S3. XRD-measurements of the used plain polymers.

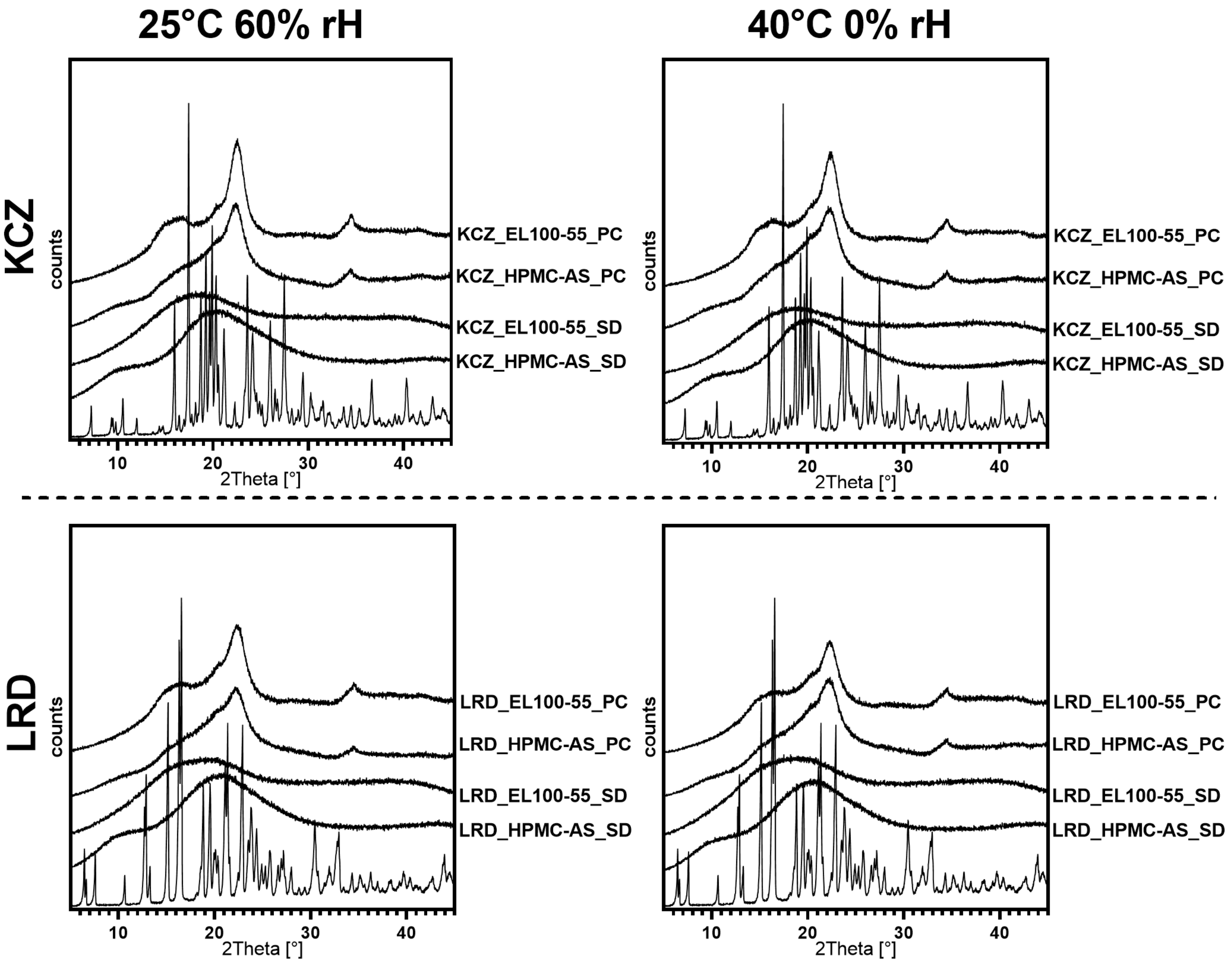

Figure S4. XRD-results of all prepared samples after storage for 6 months under different conditions (40 °C/0% rH; and 25 °C/60%rH).

Table S1. Results of the dissolution testing of all formulations showing c_{max}, t_{max}, AUC and dissolution rate determined by the linear fit prior c_{max}. The mean value is shown with the standard deviation in brackets.

Sample ID	Sieve Fraction [µm]	c_{max} [µg/mL]	t_{max} [min]	AUC	Dissolution Rate [mg/mL/min]
KCZ_HPMC-AS_SD	1000–2000	134.27 (±2.58)	30.0 (±0.00)	6,510.7 (±171.7)	5.969 (±0.025)
	710–1000	172.43 (±1.02)	25.0 (±0.00)	7,880.3 (±392.5)	10.287 (±0.174)
	500–710	183.67 (±1.26)	20.0 (±0.00)	6,677.7 (±256.0)	16.837 (±0.200)
KCZ_HPMC-AS_PC	-	182.13 (±17.14)	41.7 (±2.36)	11,828.0 (±1,530.4)	8.109 (±1.803)
KCZ_EL100-55_SD	1000–2000	121.20 (±4.24)	180.0 (±0.0)	11,767.7 (±389.4)	1.225 (±0.0)
	710–1000	177.80 (±2.69)	180.0 (±0.0)	19,042.7 (±318.1)	1.755 (±0.058)
	500–710	184.03 (±1.89)	173.3 (±9.43)	23,413.0 (±621.6)	2.639 (±0.168)
KCZ_EL100-55_PC	-	182.67 (±9.47)	180.0 (±0.0)	16,120.7 (±1,305.0)	1.497 (±0.102)
LRD_HPMC-AS_SD	1000–2000	9.90 (±0.28)	33.3 (±4.71)	1,311.7 (±81.3)	0.979 (±0.180)
	710–1000	11.60 (±0.66)	20.0 (±4.08)	1,319.0 (±225.0)	1.329 (±0.147)
	500–710	12.51 (±0.57)	15.0 (±0.0)	1,204.7 (±154.0)	1.588 (±0.226)
LRD_HPMC-AS_PC	-	11.28 (±0.16)	15.0 (±0.0)	1,117.3 (±10.9)	1.636 (±0.240)
LRD_EL100-55_SD	1000–2000	7.20 (±0.27)	53.3 (±2.36)	603.0 (±19.6)	0.199 (±0.025)
	710–1000	9.35 (±0.24)	55.0 (±4.08)	555.8 (±57.1)	0.254 (±0.015)
	500–710	12.33 (±0.73)	65.0 (±0.0)	684.8 (±55.7)	0.300 (±0.010)
LRD_EL100-55_PC	-	14.62 (±1.51)	71.67 (±2.36)	638.3 (±72.5)	0.318 (±0.055)

Table S2. Stability data: DSC-data and Karl Fischer after six months of storage under 25° C and 60% rH or 40° C and 0% rH. The mean value is shown with the standard deviation in brackets.

Sample ID	Tg [°C] 25 °C/40%—6 Month	Tg [°C] 40°C/dry—6 Month	Water Uptake [%(*w*/*w*)]
KCZ_HPMC-AS_SD	89.85 (±0.59)	88.98 (±1.13)	3.32% (±0.31%)
KCZ_HPMC-AS_PC	91.02 (±1.19)	70.88 (±1.13)	3.45% (±0.11%)
KCZ_EL100-55_SD	113.40 (±0.30)	110.83 (±1.36)	4.69% (±0.05%)
KCZ_EL100-55_PC	109.68 (±1.15)	111.85 (±2.24)	3.95% (±0.08%)
LRD_HPMC-AS_SD	86.62 (±1.62)	86.25 (±0.99)	2.83% (±0.03%)
LRD_HPMC-AS_PC	86.86 (±5.92)	88.10 (±3.83)	3.17% (±0.03%)
LRD_EL100-55_SD	114.07 (±1.20)	112.33 (±0.18)	3.91% (±0.05%)
LRD_EL100-55_PC	113.31 (±1.83)	108.89 (±1.60)	3.68% (±0.48%)
